# Tunable force transduction through the *Escherichia coli* cell envelope

**DOI:** 10.1073/pnas.2306707120

**Published:** 2023-11-16

**Authors:** Daniel P. Williams-Jones, Melissa N. Webby, Cara E. Press, Jan M. Gradon, Sophie R. Armstrong, Joanna Szczepaniak, Colin Kleanthous

**Affiliations:** ^a^Department of Biochemistry, University of Oxford, Oxford OX1 3QU, United Kingdom

**Keywords:** cell envelope, outer membrane, gram-negative bacteria, force transduction

## Abstract

The outer membrane (OM) of Gram-negative bacteria is not energised and so processes requiring a driving force must connect to energy-transduction systems in the inner membrane (IM). Tol (Tol-Pal) and Ton are related, proton motive force- (PMF-) coupled assemblies that stabilise the OM and import essential nutrients, respectively. Both rely on proton-harvesting IM motor (stator) complexes, which are homologues of the flagellar stator unit Mot, to transduce force to the OM through elongated IM force transducer proteins, TolA and TonB, respectively. How PMF-driven motors in the IM generate mechanical work at the OM via force transducers is unknown. Here, using cryoelectron microscopy, we report the 4.3Å structure of the *Escherichia coli* TolQR motor complex. The structure reaffirms the 5:2 stoichiometry seen in Ton and Mot and, with motor subunits related to each other by 10 to 16° rotation, supports rotary motion as the default for these complexes. We probed the mechanism of force transduction to the OM through in vivo assays of chimeric TolA/TonB proteins where sections of their structurally divergent, periplasm-spanning domains were swapped or replaced by an intrinsically disordered sequence. We find that TolA mutants exhibit a spectrum of force output, which is reflected in their respective abilities to both stabilise the OM and import cytotoxic colicins across the OM. Our studies demonstrate that structural rigidity of force transducer proteins, rather than any particular structural form, drives the efficient conversion of PMF-driven rotary motions of 5:2 motor complexes into physiologically relevant force at the OM.

## Significance

The outer membrane (OM) of Gram-negative bacteria is a major factor in the antimicrobial resistance crisis. The Tol system is a conserved assembly that exploits the energised inner membrane to stabilise the OM of these bacteria. System defects result in a destabilised OM and increased antibiotic susceptibility. We report the structure of the Tol motor complex, which, by comparison to other motors, supports rotation as the means of force generation. We also demonstrate that the degree of stabilising mechanical force from the motor can be modified by varying the structure of the force-transducing protein that connects the two membranes.

The asymmetric outer membrane (OM) of Gram-negative bacteria is an immobile, highly ordered, load-bearing membrane that excludes antibiotics such as vancomycin and is a virulence determinant in host–pathogen interactions (1–3). Tol (aka Tol-Pal) is a ubiquitous membrane invagination system that ensures OM stabilisation by maintaining its close connection to the peptidoglycan (PG) layer during cell division (4). To carry out this function, Tol uses cellular energy to mobilise the PG-binding lipoprotein Pal for subsequent recapture at division sites (*SI Appendix*, Fig. S1) (5, 6). However, since the OM is not energised, Tol, and its relative Ton which drives nutrient import through TonB-dependent transporters (TBDTs), relies on a motor complex in the inner membrane (IM) that is coupled to the proton motive force (PMF) (7–10). Four Tol proteins, TolQ, TolR, TolA, and TolB, together modulate the mobility of Pal (*SI Appendix*, Fig. S1). Mutations in *tol* genes cause OM destabilisation, resulting in increased detergent and antibiotic sensitivity, leakage of periplasmic contents, and reduced pathogenicity (5, 6, 10–13). The energy-dependent step in Tol function is removal of TolB from its complex with Pal in the OM, which is catalysed by TolA in the IM and energised by PMF-coupled TolQR. Energisation within Ton is similar but with a different physiological outcome. The PMF-driven motor complex ExbBD energises the IM force transducer TonB to apply force to TBDTs. TBDTs are ligand-binding OM proteins (OMPs) that utilise the force transmitted through TonB to facilitate siderophore, vitamin, and carbohydrate transport through the OM (14). Consequently, *ton* mutants exhibit reduced growth rate and virulence under nutrient-limited conditions (15–17).

The Tol and Ton motors are related to the archetypal stator unit of the bacterial flagellum, MotAB (18–22). Key residues at the interface of MotA and MotB are critical for motor function (23). These include a conserved ring of threonine residues and an aspartate involved in proton transfer, as well as proline residues within transmembrane helices, all of which are also essential in TolQR and ExbBD (22–24). Structures of MotAB and ExbBD demonstrate that an N-terminal transpore helix (TPH) of dimeric MotB and ExbD docks within a pentameric assembly of MotA or ExbB subunits, respectively (25–27). Despite both forming analogous pentameric assemblies, the monomeric topologies of MotA and ExbB differ. MotA comprises four transmembrane helices, while ExbB has only three. Additionally, MotA possesses six accessory helices, while ExbB features just three (25–27). Overall TolQ shares greater sequence similarity with ExbB than MotA (25–27).

The force transducers TolA and TonB deliver PMF-generated force from their motor complexes to the OM. Both proteins have three discernible domains. Domain I is an N-terminal transmembrane helix that interacts with the motor through a conserved Ser-His-Leu-Ser (SHLS) motif (28, 29), domain II is a structured domain that extends through the periplasm, and domain III is a conserved globular domain that interacts with the OM target by b-strand augmentation, TolB in the case of Tol and a TBDT in the Ton system (*SI Appendix*, Fig. S2) (5, 29–35). How these force transducers convert the PMF into mechanical work at the OM while traversing the cell wall is unknown (36–38). A remarkable feature of TolA/TonB is that their central domain IIs, which are typically 100 to 300 amino acids in length, are structurally dissimilar and share no sequence similarity. TolAII is an extended α-helical domain while TonBII forms a type II polyproline helix (PII) (39–42). Deletion/truncation of either domain diminishes function by reducing reach across the periplasm (43, 44), but there is little understanding of their role in communicating with the OM. We sought to understand the role of these domains in force transduction by quantitating the biological effects of a series of TolA/TonB domain II–swapped chimeras using growth assays, colicin toxicity, live-cell imaging, and OM stability tests and comparing these to a domain II deletion and a construct where domain II was replaced by an intrinsically disordered protein (IDP) domain. We also report the structure for the TolQR motor complex with which these chimeras associate. Through this combined approach, we find evidence that 5:2 motor assemblies are rotary in nature and that transduction of force from these motors to the OM depends on rigidity within the periplasm-spanning domains of force transducer proteins.

## Results

### TolQR Is a Rotameric Homologue of Other Motor Complexes.

**Fig. 1.**
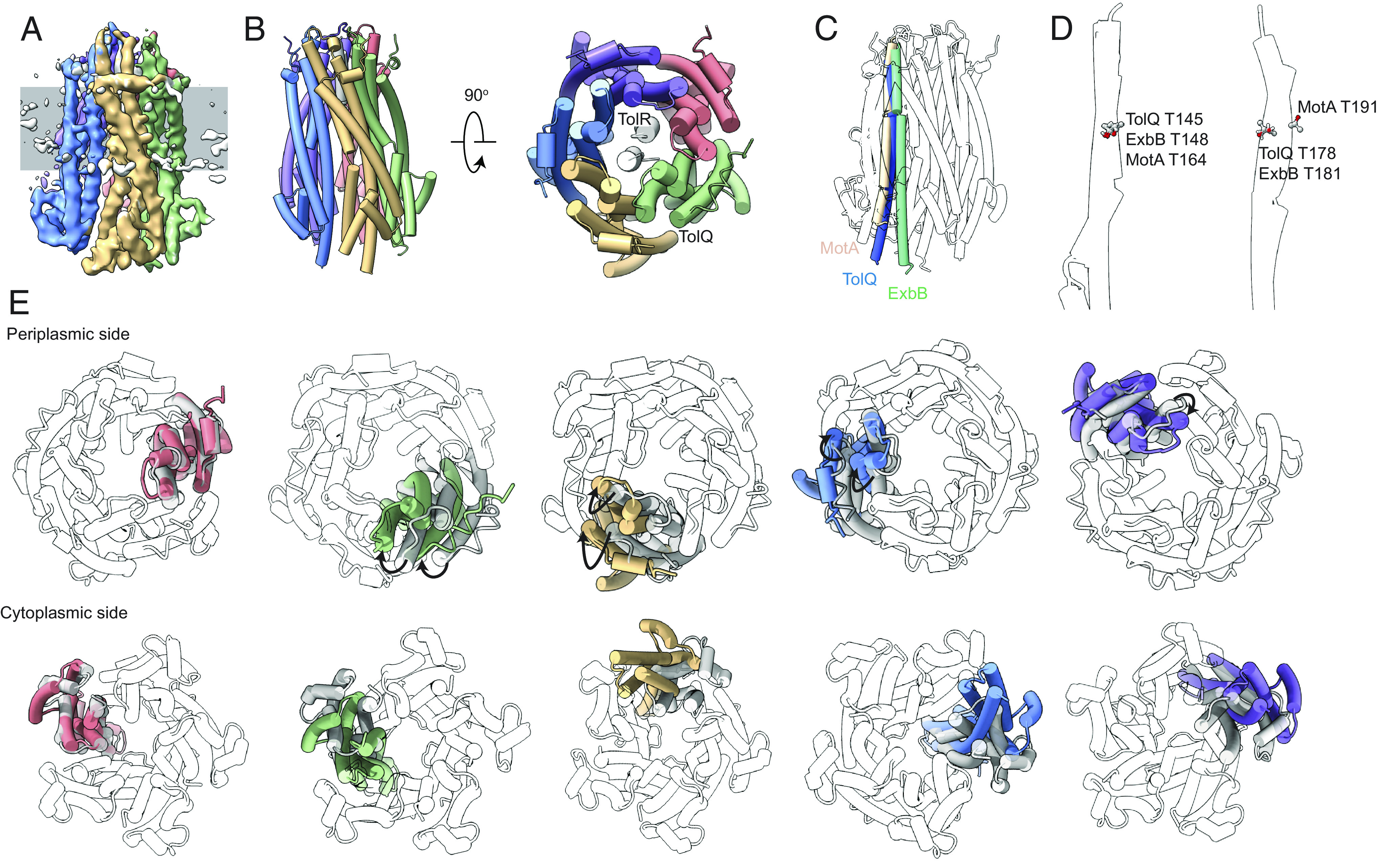
Outward rotation relates motor complex structures. (*A*) Single-particle cryo-EM map of the TolQR complex solved to 4.27 Å. Monomers are coloured individually and the position in the IM is indicated by gray rectangle. (*B*) An atomic model of TolQR was determined through rigid-body refinement into the map in (*A*). TolQ forms a pentameric structure within which a TolR (*gray*) dimer is embedded. Only the TPHs of TolR were sufficiently resolved in the cryo-EM map. (*C*) Alignment of pentameric structures of ExbB and MotA to TolQ pentamer (*transparent*), in ChimeraX. A single transmembrane helix common to ExbB (*green*), TolQ (*blue*), and MotA (*orange*) is shown which is related through clockwise rotation along the plane of the membrane. (*D*) A single chain of MotA, ExbB, and TolQ was aligned and single transmembrane helices (*transparent cartoon*) containing conserved Thr residues (*Sticks*) are shown. Conserved Thr residues are predicted to play a role in the proton transduction pathway and are accordingly situated in the same region of TM helices in all three motor complexes. (*E*) A single monomer of the ExbB (*transparent*) pentamer was aligned with the best-fitting chain of TolQ (*green*) in ChimeraX. Fit of remaining TolQ monomers (*coloured*) to ExbB (*gray*) is shown sequentially without any further realignment. Between the structures, minor perturbations of accessory helices on the periplasmic side and transmembrane helices occur, with the most pronounced distortions between the two proteins observed in the position of accessory helices on the cytoplasmic side of the pentamers.

The *E. coli* TolQR complex was isolated following affinity chromatography via a C-terminal hexahistidine tag on TolR and size exclusion chromatography (*SI Appendix*, Fig. S3 *A* and *B*). TolQR isolated in 0.01% lauryl maltose neopentyl glycol (LMNG; w/v) yielded higher order complexes in native-PAGE, within the range of 150 to 250 kDa, consistent with a TolQ pentamer (110 kDa). By contrast, the complex did not remain intact when purified in 0.02% n-dodecyl -D-maltoside (DDM) (*SI Appendix*, Fig. S3 *B* and *C*). The structure of the complex was determined to a resolution of 4.3 Å using cryo-EM (Fig. 1*A* and *SI Appendix*, Fig. S4 *A*–*C*). Rigid-body refinement of current TolQR models into the experimental map yielded an atomic representation of the complex (Fig. 1*B*) (19). The complex adopts a 5:2 stoichiometry similar to ExbBD and MotAB (Fig. 1*B*). The TPH dimer sits in the periplasmic entrance to the pentameric pore in a manner that is largely superimposable across all three assemblies. A short two-turn accessory helix (A1) forms a tight collar around the top of the hydrophobic membrane-embedded region of the pentamer, which likely stabilises interactions between the pentamer and the TPH (Fig. 1*B*). The MotA, ExbB, and TolQ pentamers all have pore diameters of approximately 30 Å on the periplasmic side, but vary considerably on the cytoplasmic side (around 40 Å, 20 Å, and 30 Å, respectively (see below)). TolQ structurally most resembles ExbB with three core transmembrane helices (T 1-3) and three accessory helices (A 1-3) (*SI Appendix*, Fig. S4 *C* and *F*). The position of conserved Thr (TolQ/ExbB/MotA) and Asp (TolR/ExbD/MotB) residues proposed to be involved in the proton transduction pathway is also structurally conserved across the three motor complexes (Fig. 1*D* and *SI Appendix*, Fig. S4*D*). Surface profiles of TolQ and ExbB pentamers are nearly identical, with the transmembrane region being similarly hydrophobic (*SI Appendix*, Fig. S4*E*) (18, 19).

The pentameric structures formed by TolQ, ExbB, and MotA are readily aligned on the periplasmic face but with perturbations related by clockwise rotation of transmembrane helices (Fig. 1 *C* and *D* and *SI Appendix*, Fig. S5). Comparison of the cytoplasmic face reveals that the TolQ pentamer adopts an expanded conformation relative to ExbB (Fig. 1*D*). An outward (into membrane) rotation of transmembrane helix 2 (T2), transmembrane helix 3 (T3), accessory helix 3 (A3), and accessory helix 4 (A4) generates this expanded pore conformation (Fig. 1*D*, *SI Appendix*, Fig. S4 *F* and *G*, and Movie S1). The difference in cytoplasmic pore diameter was predicted in a previously published Rosetta model (19), but not to the extent observed here. This expansion of the TolQ pentamer relative to ExbB is the consequence of a reduction in the total buried surface area, between pairs of neighbouring chains (*SI Appendix*, Table S1). The buried surface area between ExbB monomers ranged from 1,469 to 2,152 Å^2^, whereas TolQ exhibits weaker packing with buried surface area from 862 to 1,223 Å^2^. Finally, we find that the configuration of the TolQ pentamer is at the centre of a structural continuum of motor complexes (Fig. 1*C*). This was apparent after aligning all three structures through a single subunit, and observing that despite all three having C5 symmetry, not all subunits are equivalently arranged. Rather, rigid-body movements involving a 10 to 16° rotation of subunits from ExbB to TolQ to MotA are required to align the structures (Fig. 1*D*, *SI Appendix*, Fig. S4, and Movie S2). These comparisons emphasise that the MotAB, ExbBD, and TolQR motor complexes are rotameric as well as structural homologues, consistent with the prevailing view that these nanomachines likely generate force through rotary motion (27).

### Disordered Domain II Sustains Energised Colicin Uptake.

**Fig. 2.**
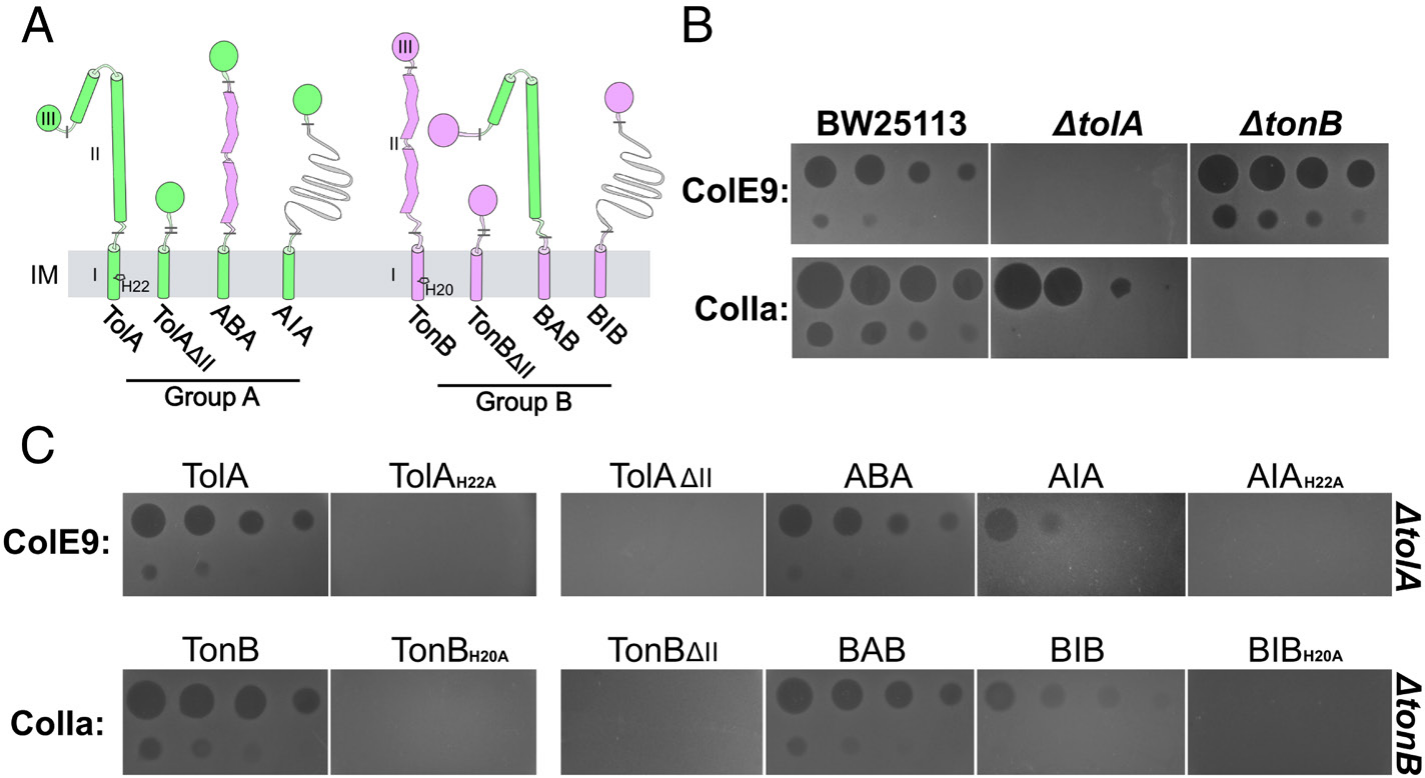
Colicin cytotoxicity assays demonstrate that domain II chimeras drive bacteriocin transport across the OM. (*A*) Domain II-swapped chimeras of TolA (*green*) and TonB (*pink*) were generated, as well as domain II knock-outs (ΔII) and substitution with an intrinsically disordered sequence of 127 amino acids (*gray ribbons*: AIA/BIB, respectively). Gray bars indicate domain swap connections. Group A constructs are defined by having a C-terminal TolAIII and group B constructs by TonBIII. The essential histidine residue of the SHLS motif within transmembrane helices of TolA and TonB (His22/20) is shown. (*B*) Controls confirming Tol and Ton dependence for colicins E9 and Ia, respectively. A fourfold dilution series of ColE9 (from 8 µM) or fivefold series of ColIa (from 10 µM) was applied to a bacterial lawn, where clearance zones indicate cell killing. (*C*) Plasmid complementation expressing wild-type TolA and TonB restores ColE9- and CoIA-mediated killing in *ΔtolA* (*Top* row) and *ΔtonB* (*Bottom* row) cells, respectively. Constructs with domain II deletions were unable to uptake colicins, but domain II swapped constructs conferred colicin sensitivity (ABA/BAB), as did constructs with disordered central domains to a lesser extent (AIA/BIB). Mutagenesis of the SHLS motif in TolA_H22A_ and TonB_H20A_ mutants abrogates killing (54–56).

Given the structural similarities of TolQR and ExbBD motor complexes, we sought to assess the interchangeability of the domain IIs of their force transducer proteins, TolA and TonB, respectively. We generated a series of partial or complete domain II–swapped TolA/TonB chimeras, where the Lys_1-2_-Ala_3-4_-(Glu/Asp) repeating sequence of TolAII was exchanged for concatenated Glu-Pro and Lys-Pro dipeptide repeat sequences of TonBII (45–47) (Fig. 2*A*). These constructs were classified as either group A (“TolA-like”) if featuring TolAIII, or group B (“TonB-like”) if featuring TonBIII. TolA and TonB domain II are both extended and structured, albeit their structures differ significantly. To test whether secondary structure itself is a requirement for force transduction, we generated an additional construct in which domain II was replaced by a 127-amino-acid IDP linker, the length of which is sufficient to span the periplasm (AIA/BIB; Fig. 2*A*). The IDP sequence was derived from an 18 amino acid repeat comprising Gly_2-4_ repeats flanked by Asn, Ser and Thr residues, from the translocation domain of colicin E9 (residues 62 to 80). This region was previously shown to be highly flexible and lacking in secondary structure by NMR (48). *E. coli* cells expressing all constructs were initially tested for functionality using colicin toxicity assays (no Tol-binding epitopes were present in the IDP linker). Colicins are bacteriocins that bind to OMPs and parasitise either TolA or TonB to energise their transport across the OM (49–51). To verify constructs were engaging with PMF-linked motors, we assessed their functionality in the context of TolA H22A and TonB H20A mutations within domain I (52, 53). As in previous studies (54–56), these mutations abolished colicin activity for both group A and group B chimeras, as did the complete truncation of domain II (Fig. 2*C*). By contrast, full-length domain II chimeras were sensitive to colicins, indicating the capacity to apply force to their respective targets in a PMF-dependent manner (Fig. 2*C*). A range of additional TolA/TonB chimeric constructs varying in domain II length and composition, as well as domain I exchanged variants, were all susceptible to colicins (*SI Appendix*, Fig. S6).

Remarkably, AIA and BIB were colicin sensitive, and this activity was demonstrably dependent on motor interactions. We conclude that a structured domain II is not a prerequisite for force transduction between the two membranes by either Tol or Ton, but loss of domain II secondary structure diminishes colicin sensitivity.

### Group A Chimeras Vary in Their Propensity to Stabilise the OM.

**Fig. 3.**
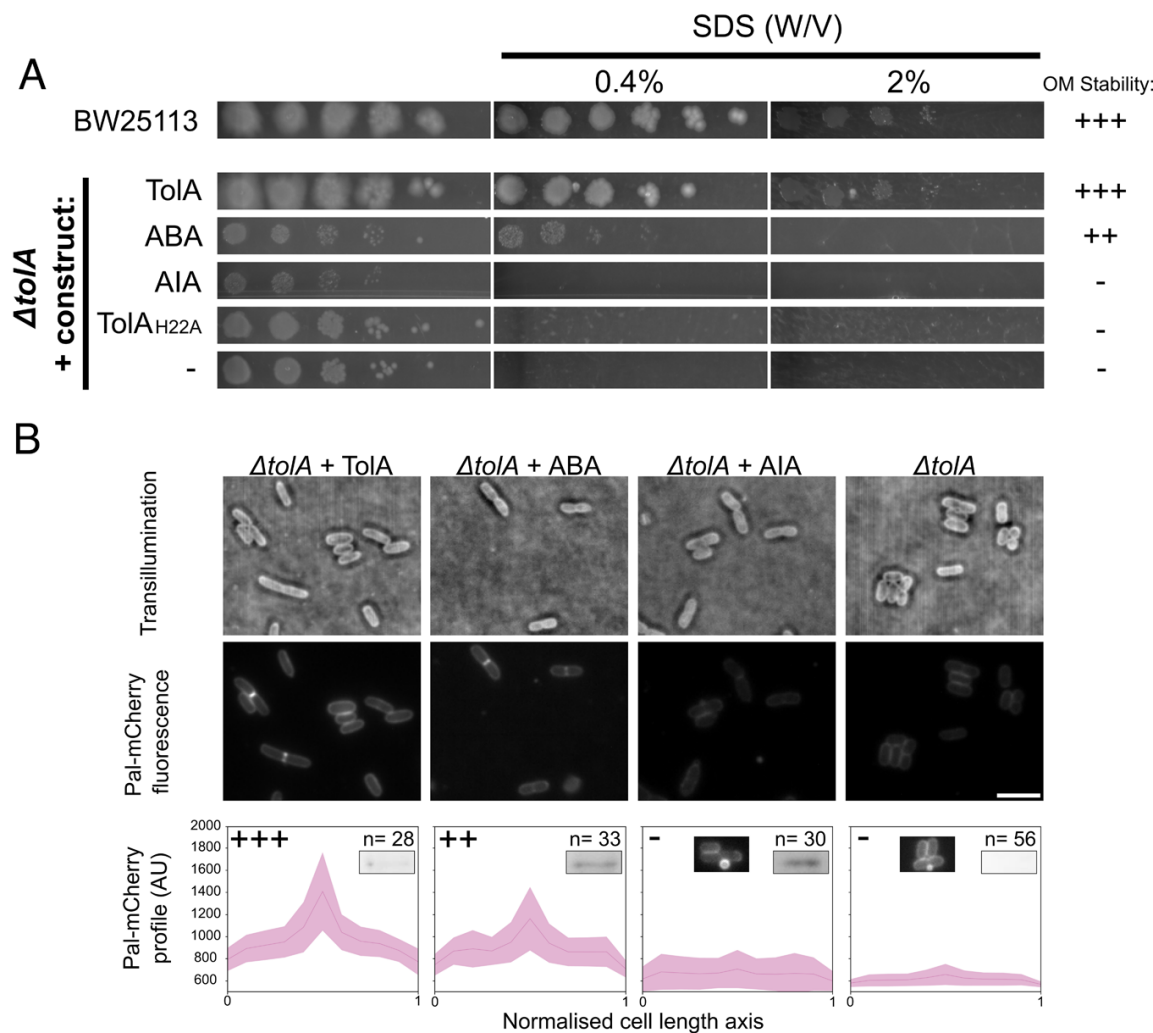
OM stabilisation by Tol requires a structured TolA domain II. (*A*) Group A constructs demonstrate a continuum of OM stability. Full complementation (wild-type growth), on 2% SDS (w/v) graded “+++”, moderate growth on 0.4% SDS (w/v) rated “++”, and low growth on 0.4% SDS rated “+”, in accordance with additional mutants which demonstrated intermediate SDS-tolerance (*SI Appendix*, Fig. S8*A*). (*B*) Group A constructs exhibit a spectrum of Pal accumulation, which correlates with observed OM stability. *ΔtolA Pal-mCherry* cells were transformed with group A construct expression plasmids, grown to mid-log phase, and then imaged by transillumination (*Top*) and fluorescence microscopy (*Centre*). (The scale bar indicates 5 µm.) Fluorescence profiles of dividing cells were obtained by plotting medial fluorescence along the cell length axis, which was then averaged per construct type (*Bottom*). The central line indicates the mean fluorescence value along the cell length axis, and shaded bands indicate ± SD. Relative SDS tolerance levels from panel A are indicated *Top Left*, and number of dividing cells averaged per profile is also indicated *Top Right*. (*Inset*) Western blot bands indicate the expression levels of constructs (*SI Appendix*, Fig. S9), and micrographs indicate vesiculation of *ΔtolA* cells ± AIA.

We next asked how effective domain II chimeras were in terms of supporting endogenous function, which comprises nutrient uptake for Ton and OM stabilisation for Tol. All group B constructs supported growth in minimal media (*SI Appendix*, Fig. S7), including BIB, but the phenotypes were not sufficiently discerning to draw any mechanistic conclusions. By contrast, the effectiveness of group A chimeras in stabilising the *E. coli* OM was more illuminating. OM stability was assessed in two ways: resistance against varying concentrations of the surfactant sodium dodecyl sulfate (SDS) and accumulation of Pal at septa, which signals effective invagination of the OM during cell division (Fig. 3). Unlike colicin overlay assays (Fig. 2), where most chimeras behaved similarly, these assays exhibited phenotypes that ranged from near wild-type to being inactive. For example, the group A chimera ABA, in which domain II from TolA was switched for domain II from TonB, could grow on 0.4% SDS (w/v) agar plates but not on 2% SDS, as can cells expressing wild-type TolA, indicative of partial OM stabilisation. Similar partial OM stabilisation was observed for additional chimeras where domain II was further elongated (AABA and ABBA; *SI Appendix*, Fig. S8*A*). AIA however could not stabilise the OM, its growth characteristic on plates being essentially indistinguishable from those of the *tolA* deletion or the TolA_H22A_ mutant that abolishes association with the motor.

A more discerning indicator of Tol function for group A chimeras was their ability to accumulate Pal at division septa. For fluorescence-based experiments, plasmids expressing chimeric constructs were transformed into a *ΔtolA* mutant strain with a chromosomal *pal-mCherry* fusion gene, and Pal-mCherry accumulation was examined using fluorescence microscopy, as previously described (Fig. 3*B* and *SI Appendix*, Fig. S8*B*) (5, 6, 11).

These experiments indicated that the degree of septal Pal accumulation correlates positively with the degree of OM stabilisation observed in SDS assays, which is also seen for other group A constructs of varied domain I and II structure/length (*SI Appendix*, Fig. S8). Western blots confirmed that this variation in stability between group A constructs was not due to differences in protein abundance (*SI Appendix*, Fig. S9). To test whether Pal distributions were altered in response to mislocalisation of TolA, fluorescence microscopy of the green fluorescent protein (GFP)-tagged TolA constructs was also assessed. These demonstrated similar expression levels and septal localisation (*SI Appendix*, Fig. S10). We conclude that OM stabilisation by Tol is a tunable phenotypic trait in *E. coli* that becomes evident in strains expressing chimeric TolA constructs carrying alternate domain IIs and that the degree of OM stabilisation correlates with the degree of Pal accumulation at division septa.

### Colicin-Import Kinetics Mirror the Efficiency of Tol-Dependent OM Stabilisation.

**Fig. 4.**
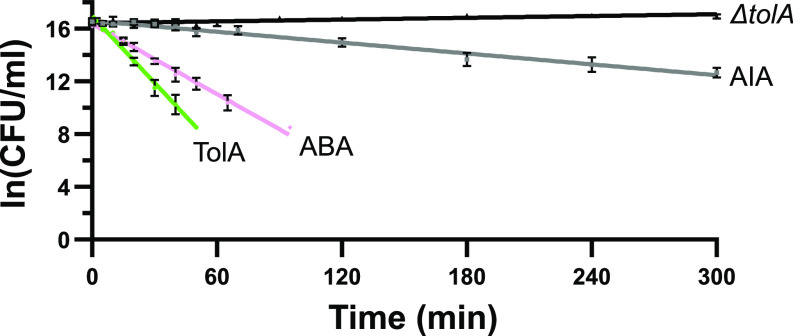
Colicin transport across the OM correlates with the efficiency of force transduction mediated by TolA domain II. Time-kill assay shows rate of uptake varies with domain II structure and appears to decrease as a consequence of intrinsically disordered domain II. Cells were treated with 100 nM ColE9 and incubated in LB media at 37 °C with shaking; then, aliquots were incubated with trypsin at indicated time points. The number of colony-forming units (CFU) was used to assess the number of live cells in suspension, where the decrease in viable cells over time reflects the rate of cell killing. Mean ColE9 translocation half-lives for different constructs were calculated from the logarithmic slope, as 4.1 min (TolA), 7.8 min (ABA), and 50.4 min (AIA). 95% CIs of mean translocation half-life were 3.7 to 4.6 min (TolA), 7.2 to 8.5 min (ABA), and 44.3 to 58.5 min (AIA). N = 3 biological replicates per sample, each comprising 3 technical repeats. Error bars indicate the SEM.

The functional characterisation of TolA chimeras highlighted a contradiction; AIA, in which a disordered sequence replaces the structured central domain II of TolA, was functional for ColE9 import but was not capable of stabilising the OM (Figs. 2 and 3). Closer inspection of the ColE9 data suggested that AIA-expressing cells were less sensitive than other chimeras (reduced number of killing zones). Given the long time course of these experiments (*E. coli* overlay cultures on agar plates are incubated overnight with serial dilutions of the bacteriocin), we performed time-resolved ColE9 killing assays at a single concentration (100 nM) to compare the rates of colicin-mediated toxicity for TolAII chimeras. Previous work has shown that this assay, in which ColE9 import is halted by trypsin treatment, reports on the rate-limited transport of colicins across the OM (57, 58). ColE9 translocation half-lives calculated from the resulting pseudo-first-order plots for TolA, ABA, and AIA were 4.1, 7.8, and 50.4 min, respectively (Fig. 4). The data for wild-type TolA are similar to those reported previously (58), whereas those for AIA-expressing cells are an order of magnitude slower. This rate improves six-fold for ABA, which contains the structured domain II from TonB, but even this construct is two-fold less effective at transporting ColE9 across the OM than wild-type TolA. We conclude that the relative impact of group A Tol chimeras on ColE9 import kinetics mirrors their effects in septal Pal localisation and OM stability. These data also imply that differing levels of force are delivered to the OM via these chimeras that have distinct physiological outcomes. Worst affected is AIA that imports ColE9 poorly and cannot stabilise the OM. ABA is significantly better at importing ColE9 and can partially stabilise the OM, but only the helical domain II of TolA provides full OM stabilisation and imports ColE9 with greatest efficiency.

## Discussion

**Fig. 5.**
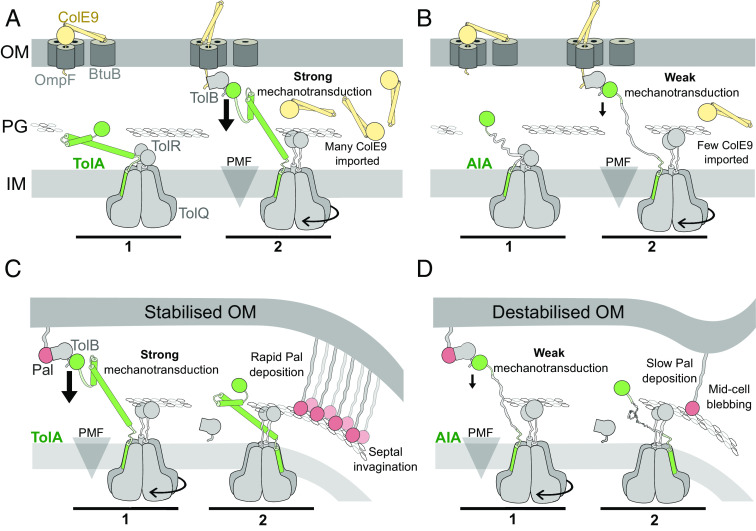
Efficient force transduction to the OM for Pal-mediated OM stabilisation and invagination requires secondary structure in TolAII. (*A*) TolA rapidly imports colicins. I) TolA engages TolQR below the PG layer. 2) TolQR energises conformational rearrangements within TolAII to apply tension to the TolB-ColE9 complex (large arrow) and entry of unfolded ColE9 to the periplasm from where it refolds and translocates to the cytoplasm to cause cell death through nuclease action (51). The precise conformational changes that take place during force transduction are currently unknown but may, for example, involve a combination of TolA refolding and/or wrapping about the stem of TolR, together transducing a torsional force perpendicular to the OM. (*B*) AIA imports colicins slowly. 1) AIA interacts with TolQR. 2) Force transduction via AIA occurs less efficiently than via TolA (small arrow). This is sufficient to import some cytotoxic colicins, but their passage across the OM is significantly slower than in wild-type TolA cells. (*C*) TolA rapidly recruits Pal to the division site, stabilising and invaginating the OM at the septum. I) TolQRA is trafficked to the division septum to capture mobilised TolB-Pal complexes. 2) TolA efficiently dissociates TolB-Pal complexes, depositing many Pal monomers which stabilise the OM. (*D*) AIA exhibits poor Pal recruitment at the septum, which fails to stabilise the OM. 1) AIA is a weak mechanotransducer in the forced dissociation of the TolB-Pal complex. 2) The inefficiency of AIA force transduction slows Pal accumulation at the septum, resulting in OM destabilisation and blebbing.

Rotary motors are essential membrane-embedded biological nanomachines. A seminal example of experimental evidence for motor rotation, is the F_1_F_0_ ATP synthase complex where proton flux drives synthesis of ATP from ADP and inorganic phosphate (59, 60). While the asymmetric 5:2 assemblies of MotAB, ExbBD (Ton), and now TolQR nanomachines in bacteria strongly imply these are rotary motors, this has yet to be demonstrated experimentally. In all cases, a pentameric assembly in the cytoplasmic membrane forms a protein pore within which the transpore helices of dimeric MotB, ExbD and TolR, respectively, reside. Overlays of the cryo-EM structures for all three motor complexes reveal that a clockwise rotation relates the monomeric units of the pentamer. We suggest these structural differences reflect the rotatory nature of these complexes, which in vivo would be driven by proton flow through the pentamer assemblies involving conserved protonation sites, as discussed previously (18, 19). A further requirement for motor function is a stator against which rotation can occur. Unlike the F_1_F_0_ where protein subunits act as stators, this role is taken by the dimeric subunits of MotB, ExbD, and TolR binding the cell wall (Fig. 5) (61–63). In most instances, this is inferred since there have been few studies showing stator binding to PG and in all cryoEM structures of these 5:2 assemblies (including TolQR) the PG-binding domains are not resolved.

A fundamental difference between the rotary nanomachines of Ton and Tol and that of Mot is the directionality of force transduction. Unlike Mot, where the force generated by stator units is delivered to the flagellar rotor directly, Ton and Tol motors deliver this force perpendicularly to the OM through their force transducer proteins, TonB and TolA, respectively. This requires the force transducers to associate with motors, traverse the cell wall, and span the periplasm to contact OM targets. Associations with their respective motors as well as with OM targets are conserved, suggesting a conserved force transduction mechanism is at play (*SI Appendix*, Fig. S2) (24, 54). Yet the identity of the intervening domain II of the proteins that delivers this force is divergent. The present work highlights two important principles about the involvement of this domain in force transduction.

First, spanning the width of the periplasm with a disordered polypeptide does not propagate enough force to carry out TolA’s endogenous function of OM stabilisation but is sufficient to import cytotoxic colicins across the OM (Fig. 5). The force required to pull unfolded ColE9 through its porin translocator OmpF is presumably similar to that used to dissociate TolB from Pal as they are both mediated by the TolB-TolA complex, where a TolB β-strand is added to a TolA β-sheet, against which force is applied perpendicular to the plane of the OM (*SI Appendix*, Fig. S2*B*). Since very few imported ColE9 molecules are needed to kill a cell, even low-efficiency mutants such as AIA can effectively import colicins without imparting OM stabilisation. Hence, colicin uptake (and the force transduction involved) does not equate to OM-stabilisation, as suggested previously (64). Rather, full complementation of a *tolA* deletion is contingent on sufficient PMF-dependent force being transduced from the TolQR motor to enable thousands of Pal molecules to be accumulated at the division site thereby invaginating the OM (5, 6, 65).

Second, for physiologically relevant force to be transduced from the motor to the OM requires only a structured domain II. This requirement likely explains why very different domain IIs exist in diverse force transduction systems in Gram-negative bacteria, including type 9 secretion and gliding motility (66, 67). Nevertheless, the nature of the intervening structure has an impact on the efficiency of force transduction, as evidenced by group A chimeras where increasing segments of TonB grafted into TolA (ABA, AABA, and ABBA) result in defective Pal localisation at division sites, diminished OM stabilisation, and slower colicin uptake (Figs. 2 and 3 and *SI Appendix*, Figs. S6 and S8).

## Materials and methods

### Bacterial Strains and Conditions.

Competent cells were generated by calcium chloride chemical competency protocol and stored at –80 °C (68). Inoculants were thawed, precultured in high salt lysogeny broth (LB), transformed by 45-s heat shock (69), and plated onto appropriate LB agar selection plates before storage at 4 °C. Precultures were grown overnight in 5 ml LB. Media were supplemented with ampicillin [100 μg/mL (w/v)], chloramphenicol [35 μg/mL (w/v)], or kanamycin [50 μg/mL (w/v)], depending on selection markers. Cells to be imaged were precultured to stationary phase and subcultured to mid-log phase in 5 mL M9 media. A *ΔtolA::kan* strain (BW25113 background) was used to test TolA-like constructs, and a *ΔtonB::kan* mutant was used to test TonB-like constructs (70). Strain genotypes are indicated in *SI Appendix*, Table S4.

### Construction of Chimeras and Mutants.

Plasmids were designed by a combination of techniques (whole plasmid mutagenesis, excision/ligation, and Gibson assembly) (Table 1). All TolA and TonB mutant constructs were expressed in pBAD24, which gives a basal expression close to wild-type levels in *ΔtolA* or *ΔtonB* cells in the BW25113 background strain (65, 71). Plasmid sequences with their relevant primers are indicated in supplementary folder “plasmids.zip” with an accompanying text file. The IDP sequence was cloned from 3× 18 amino acid repeats adapted from a flexible loop of *colE9* and did not include the TolB-binding epitope (72). All TolA/TonB constructs shown feature an N-terminal GFP fusion protein. The *gfp* gene was cloned from pQE-60NA-msGFP2, which was a gift from Benjamin Glick (Addgene plasmid # 135301; http://n2t.net/addgene:135301; RRID:Addgene_135301) (73).

**Table 1.** Plasmids used in this study

Name	Genotype	Abbreviation	Reference
pCEP13	pT12-*tolQ-tolR*	N/A	Deme et al. (25)
pJS61	pBAD24-GFP-*tolA*	TolA	Connolley et al., (65)
pJS70	pBAD24-GFP-*tolA* (H22A)	TolAH22A	This study
pJS65	pBAD24-GFP-GGGGS-*tonB*(2-239)	TonB	Valbuena et al., (73)
pJS96	pBAD24- GFP-GGGGS-*tonB* (H20A)	TonBH20A	This study
pJS104	pBAD24-GFP-*tolA*(1–47)*-tolA*(311–421)	TolAΔII	This study
pJS109	pBAD24-GFP-*tolA*(1–45)-*tonB*(33–168)-*tonB*(33–168)-*tolA*(311–421)	ABBA	This study
pJS110	pDWJ26 (H22A)	AIAH22A	This study
pJS119	pBAD24-GFP-*tonB*(2–40)-*tonB*(168–239)	TonBΔII	This study
pJS124	PDWJ27 (H20A)	BIBH20A	This study
pJMG1	pBAD24-GFP-GGGGS-*tonB*(2–62)-*tonB*(105–239)	TonBΔPII	This study
pJMG2	pBAD24-GFP-GGGGS-*tonB*(2–62)-*tolA*(48–310)-*tonB*(105–239)	BAB	This study
pDWJ13	pBAD24-GFP-*tolA*(1-320)-*tonB*(139–239)	AAB	This study
pDWJ14	pBAD24-GFP-*tolA*(1-220)-*tonB*(39–239)	AABB	This study
pDWJ15	pBAD24-GFP-*tonB*(2-139)-*tolA*(321–421)	BBA	This study
pDWJ16	pBAD24-GFP-*tonB*(2–45)-*tolA*(46–421)	BAA	This study
pDWJ17	pBAD24-GFP-*tolA*(1–45)-*tonB*(46–239)	ABB	This study
pDWJ18	pBAD24-GFP-*tolA*(1–45)-*tonB*(33–168)-*tolA*(311–421)	ABA	This study
pDWJ22	pBAD24-GFP-*tolA*(1-220)-*tonB*(39–139)-*tolA*(311–421)	AABA	This study
pDWJ26	pBAD24-GFP-*tolA*(1–45)-IDP129*-tolA*(311–421)	AIA	This study
pDWJ27	pBAD24-GFP-*tonB*(2–40)-IDP129*-tonB*(139–239)	BIB	This study

Plasmids available from Addgenes (77) and corresponding author.

Plasmid pJS65 includes *gfp* gene cloned from ref. 73.

### Protein Production and Purification.

*E. coli* TolQR was prepared by chemical transformation of the expression plasmid (pCEP13), encoding TolQ and TolR in a modified pT12 backbone into BL21 (DE3) cells (New England Biolabs). Vector modification involved introduction of a hexahistidine tag following the C-terminal twin-step tab. Proteins were coexpressed in TB media supplemented with kanamycin (50 µg/mL), rhamnose (0.1% w/v) and day culture added to achieve an OD_600_ of ~0.02. Cells were maintained at 37 °C overnight (~18 h) before harvesting via centrifugation. Pelleted cells were resuspended in 50 mM Tris/HCl pH 7.5, 500 mM NaCl, 5 mM imidazole, and 1 mM PMSF and lysed by sonication. The soluble supernatant following low-speed centrifugation (10,000 × g, 4 °C, 15 min) was subjected to ultracentrifugation (200,000 × g, 4 °C, 45 min) to pellet total membrane fraction. The total membrane pellet was then solubilised in 50 mM Tris/HCl pH 7.5, 500 mM NaCl, 5 mM imidazole, and 1% (w/v) LMNG at 4 °C overnight. After solubilisation the remaining insoluble fraction was pelleted by ultracentrifugation (200,000 × g, 4 °C, 45 min). The soluble supernatant was then applied to nickel resin on a gravity flow column. TolQR complexes were bound to the nickel resin through affinity to a genetically encoded His-tag on TolR and were washed with buffer containing 50 mM Tris/HCl pH 7.5, 500 mM NaCl, 5 mM imidazole, and 0.02% (w/v) LMNG. TolQR was eluted with buffer containing 50 mM Tris/HCl pH 7.5, 500 mM NaCl, 500 mM imidazole, and 0.02% (w/v) LMNG. The purified protein was buffer exchanged using a Superdex 200 16/600 PG chromatography column equilibrated with size exclusion chromatography (SEC) buffer containing 50 mM Tris/HCl pH 7.5, 300 mM NaCl, 2 mM ethylenediaminetetraacetic acid (EDTA), and 0.01% (w/v) LMNG. Protein was concentrated in a 100 kDa molecular weight cut-off (MWCO) spin concentrator, and concentration was calculated from absorbance at 280 nm assuming a sequence-based extinction coefficient of 36,420 M^-1^cm^-1^.

ColE9 and ColIa were purified from IPTG-induced cultures of BL21 (DE3) by Ni-affinity chromatography and size exclusion chromatography as previously described (50, 72). ColIa was lyophilised and stored at –20 °C previously by NGH, which was solubilised here in phosphate-buffered saline pH 7.2.

### Single-Particle Cryo-EM Data Collection.

TolQR complex in SEC buffer was applied to Quantifoil 300 mesh Cu, 1.2/1.3, grids at a concentration of 7.5 mg/mL. Samples (3 mL) were vitrified using a Vitrobot Mark IV (Thermo Fisher) with a blot time of 3 s, blot force of 10, at 8 °C with 100% humidity. Electron micrographs were acquired on a 300-kV Titan Krios microscope, equipped with a K2 detector (Gatan), recording in counting mode. A pixel size of 0.822 Å was used to collect videos of 30 frames with a total dose rate of 46.39 electrons/Å^2^. For further collection parameters, see *SI Appendix*, Table S3.

### Cryo-EM Data Processing and Model Building.

All processing steps were carried out in cryoSPARC (74). Patch motion correction and contrast transfer function (CTF) estimation were carried out on all 17,494 movies after which 25 were excluded to give 17,469 images for further processing. Initially, a subset of micrographs (8,500) was blob picked to generate templates for picking. Following picking, particles were sorted by multiple rounds of two-dimensional (2D) classification and nonuniform refinement to generate improved templates with a variety of views for more specific particle picking. Particles were repicked with high-resolution templates, from which 7,233,763 particles were extracted with a 208-pixel box. These were sorted, and aligned with 2× rounds of 2D classification, followed by *ab initio* model generation (3 classes) and nonuniform refinement. To generate a higher-resolution model, particles (1,313,589) were extracted in a larger box (300 pix) and sorted again using 2D classification. 566,482 particles were subsequently non-uniformly refined, and the resulting map (4.51 Å) was locally refined using a static mask to exclude the majority of the detergent belt, generating a map at 4.27 Å resolution. Particles were then sorted using a 3D classification job and a single class (114,830 particles) with clear TolR density further refined using local refinement to generate a model at 4.7 Å. Processing is summarised in *SI Appendix*, Fig. S11. Docking of the TolQR model (19) into maps was unambiguous. Due to the resolution of the map, only rigid body refinement was run in Phenix (75).

### Blue Native PAGE.

Protein samples (9 µL) were made up at 23 µM in buffer containing 50 mM Tris/HCl pH 7.5, 300 mM NaCl, and either 0.01% LMNG or 0.02% DDM as appropriate (w/v). 5% G250 (Thermo Fisher Scientific, BN2004) was added to achieve a final concentration of 0.008% in the sample. Gels were run and stained as described previously (76).

### Killing Assays.

For plate-based assays, high salt LB agar selection plates were cast. Then, 100 μL mid-log cell culture (OD_600_: 0.6) grown in LB was resuspended in 6 mL soft-LB agar [0.7% agar (w/v)] and cast onto the surface of the selection plate. After setting, colicins were applied (1 μL spots) in a dilution series. ColE9 was spotted on in a fourfold dilution series starting with 8 μM, and ColIa was spotted on in a fivefold dilution series starting from 10 μM. Plates were incubated overnight at 37 °C. For time-kill kinetics assays, cells were grown to OD 0.4; then, growth was stalled with chloramphenicol (20 µg/mL). After 2 min of incubation, 150 µL was taken and treated with 100 nM ColE9; then, cells were incubated at 37 °C with shaking at 500 RPM. At each sample collection time point, 1 mg/mL trypsin was added to 10 µL cells and incubated for 30 min in the conditions described. Translocation half-lives were calculated from slope (decay constant) of the linear regression of colony-forming units/time semi-log plot. Protocol was adapted from Francis et al. (58). No induction was used for plate-based or liquid killing assays, except BAA (*SI Appendix*, Fig. S6) which was induced with 0.02% arabinose (w/v) due to poor expression (*SI Appendix*, Figs. S9 and S10).

### SDS Assays.

Cells were grown to OD_600_ 0.5 in LB, with induction from the point of inoculation for low-expression mutants. A tenfold dilution series was prepared in LB with the corresponding selection antibiotics. The series was then applied (in 1 µL spots) onto LB agar plates containing 0, 0.4, or 2 % SDS (w/v), with no antibiotics for selection. Additional plates with a tenfold series of arabinose concentrations [0.002 to 2% (w/v)] were used to identify optimal Δ*tolA* complementation between different mutants (Table 1).

### Fluorescence Microscopy and Analysis.

*ΔtolA Pal-mCherry* cells were transformed with group A construct expression plasmids then grown to OD_600_ 0.5 in M9 minimal media with appropriate selection antibiotics. Cells were pelleted and then resuspended in a small volume of fresh M9 and 4 µL cells pipetted onto agarose (1% w/v), prepared in M9 media. Agarose pads were set on glass slides by pipetting molten M9-agarose onto the glass within the Geneframe. After the agarose had set, resuspended cells were applied and a glass coverslip pressed on top, to adhere to the GeneFrame adhesive. Cells were imaged using the Oxford Nanoimager-S with a 100×/1.49 oil immersion objective lens and a pixel size of 117 nm. Each frame had a 100-ms acquisition time. Imaging of Pal-mCherry and GFP-mutant constructs was performed in two sequential fluorescence excitation steps. Cells were imaged with 650 nm transillumination for 100 frames, then 560 nm excitation of Pal-mCherry for 100 frames, then 473 nm excitation of the corresponding GFP-construct for 100 frames, to minimise fluorophore bleaching. Cells were imaged at a 49.5° angle to reduce background fluorescence (78), and both lasers were used at 10% power for excitation. During image analysis, the first 50 frames were averaged as a representative transillumination image, and the first 10 frames from the point of each fluorescence activation were averaged as a representative fluorescence image. Cells were segmented within MicrobeJ (79). Segmented cells were manually labelled as elongating or dividing for subsequent profile analyses. Profile analyses compare dividing cells automatically normalised by length, plotted against mean fluorescence per pixel along the segmentation-determined cell length axis. Fluorescence micrographs are shown with equivalent brightness and contrast settings.

### Western Blots.

Cell pellets of 0.5 mL of cell culture normalised to OD_600_ 0.5 were harvested and stored at –20 °C. Cells were lysed by boiling in 1× SDS buffer for 20 min. Whole cell lysates were resolved by SDS-PAGE on 12% polyacrylamide gels, along with ECL rainbow ladder (Amersham). Proteins were transferred onto PVDF membrane by PowerBlot for 45 min at 45 mA. Membranes were blocked overnight (α-TolA) or 1 h (α-GFP) at room temperature in tris-buffered saline with 0.1% Tween® 20 Detergent (TBST) buffer with 8% milk powder (w/v). Membranes were then washed and labelled with either α-GFP (1:1,000, overnight at room temperature) or α-TolA (1:1,000, 1 h at room temperature) primary antibodies with 4% milk powder in TBST (65). Membranes were then washed with TBST and labelled for 1 h with secondary anti-rabbit antibody conjugated with horseradish peroxidase (1:1,000) in the presence 4% milk powder in TBST. Membranes were washed again and ECL chemiluminescence kit was applied, and membranes were imaged 1 min after development.

### Growth Curves.

Cells expressing group B constructs were grown overnight at 37 °C in M9 media with shaking, with selection antibiotics where appropriate. Inoculants were prepared by diluting overnight samples to OD_600_ = 0.1 in M9 media. From the diluted cultures, 10 µL was used to inoculate 190 µL M9 media aliquotted within a 96-well plate. The 96-well plate was incubated at 37 °C with shaking, and optical densities were recorded at 630 nm on an Accuris SmartReader 96.

## Supplementary Material

Appendix 01 (PDF)

Movie S1. **Structural morph from ExbB pentamer to TolQ pentamer.** ExbBD (6TYI) chain IDs were renamed to match TolQR nomenclature and pentameric structures aligned on chain A within ChimeraX (matchmaker). Structural morph was then generated to track transition required to get from ExbB (start) to TolQ (end).

Movie S2. **Structural morph from ExbB pentamer to TolQ pentamer to MotA pentamer.** ExbBD (6TYI) and MotAB (6YSL) chain IDs were renamed to match TolQR nomenclature and pentameric structures aligned on chain A within ChimeraX (matchmaker). Structural morph was then generated to track transition required to get from ExbB (start) to TolQ (middle) to MotA (end).

## Data Availability

Raw data available in Zenodo repository (killing assays, microscopy, cell counts) (80). Protein structure and associated data have been deposited in Protein Data Bank and Electron Microscopy Data Bank (80DT and EMD-16816) (81, 82). All other data are included in the article and/or supporting information. Plasmids available from Addgene (https://www.addgene.org/plasmids/articles/28238657/) (77) and corresponding authors.
