# Supplementary material for: Tunable force transduction through the Escherichia coli cell envelope
Source: Proc Natl Acad Sci U S A. 2023 Nov 16;120(47):e2306707120. doi: 10.1073/pnas.2306707120 (PMC10666116; doi:10.1073/pnas.2306707120)
Supplement: Supplementary file 1 — Appendix 01 (PDF) [file pnas.2306707120.sapp.pdf]

Supplementary Table 1: Buried surface area between neighbouring monomers within TolQ and ExbB pentamers

Chain 1	Chain 2	Buried SA TolQ (Å^3)	Buried SA ExbB (Å^3)
A	B	966	1570
B	C	862	2152
C	D	1208	1622
D	E	1223	1692
E	A	1149	1469

Buried surface area was a calculated using ChimeraX.

Supplementary Table 2: Degrees of rotation between matching rotamers of TolQ and ExbB

TolQ reference chain	ExbB matched chain	Rotation (º)
A	A	0.4
B	B	10.8
C	C	16
D	D	14
E	E	10

ExbB was aligned to TolQ through chain A, the degree of rotation between the subsequent rotamer matches (defined as chains that were overlaid on top of each other) was calculated in Pymol using draw_rotation_axis.py script

Supplementary Table 3: Cryo-EM Data collection

	TolQR<br>EMD-16816<br>PDB-8ODT
**Data collection**	
Microscope	Titan Krios (Cosmic)
Voltage (kV)	300
Detector	Gatan K3
Recording mode	Counting
Magnification	190,000
Movie/micrograph pixel size (Å)	0.822
Dose rate (e-/Å^2/sec)	4.8
Number of frames per movie	30
Movie exposure time (s)	8
Total dose (e-/Å^2)	46.39
Defocus range (um)	-0.5 to -2
**EM data processing**	
Number of movies/micrographs	17,496
Box size (px)	300
Particle number (total)	7,233,763
Particle number (post 2D)	1,313,589
Particle number (post 3D)	566,482
Particle number (used in final map)	114,830
Symmetry	C1
Map resolution (FSC 0.143)	4.2
Local resolution range (FSC 0.5)	4.0-6.1
Map sharpening B-factor (Å^2)	-225
Initial model used	Webby *et al.*, 2022 (1)
Model composition	
Non-hydrogen protein atoms	18800
Protein residues	1188
RMSD from ideal	

Bond length (Å)	0.006
Bond angles (°)	0.709
Validation	
Molprobity score	2.31
Clashscore	16.6
Rotamers outliers (%)	3.61
CC model-vs-map (masked)	0.66
Ramachandran plot	
Favoured (%)	97.10
Allowed (%)	2.81
Outliers (%)	0.09

Supplementary Table 4: Strains used in this study

Strain name	Abbreviation	Genotype	Usage	Reference
BL21 (DE3)		F- *ompT hsdSB(rB–, mB–) gal* dcm	Protein purification	(2)
DH5α		F- *endA1 glnV44 thi-1 recA1 relA1 gyrA96* deoR nupeptidoglycan Φ80dlacZΔM15 Δ(*lacZYA- argF*)U169, *hsdR17*(rK- mK+), λ–	Cloning	(3)
BW25113		F-, Δ(*araD-araB*)*567* Δ(*rhaD-rhaB*)*568 ΔlacZ4787* (::rrnB-3) *hsdR514 rph-1*	WT controls	(4)
JW0729-3	*ΔtolA*	F-, *Δ(araD-araB)567, ΔlacZ4787(::rrnB-3), ΔtolA788::kan,λ-, rph-1, Δ(rhaD-rhaB)568, hsdR514*	Group A constructs expression	(5)
RKCK10	*ΔtolA Pal-mCherry*	F-*, Δ(araD-araB)567, ΔlacZ4787(::rrnB-3), ΔtolA788::frt, λ-, rph-1, Δ(rhaD-rhaB)568, hsdR514 pal::pal-GGGGS-mCherry(ΔAgeI)(ΔEcoRI)::kan*	Group A constructs expression	(6)
JW5195-1	*ΔtonB*	F-, *Δ(araD-araB)567, ΔlacZ4787(::rrnB-3), ΔtonB760::kan, λ-, rph-1, Δ(rhaD-rhaB)568, hsdR514*	Group B constructs expression	(5)

Plasmid maps of constructs are indicated in the supplementary folder “plasmids.zip” with an accompanying text file.

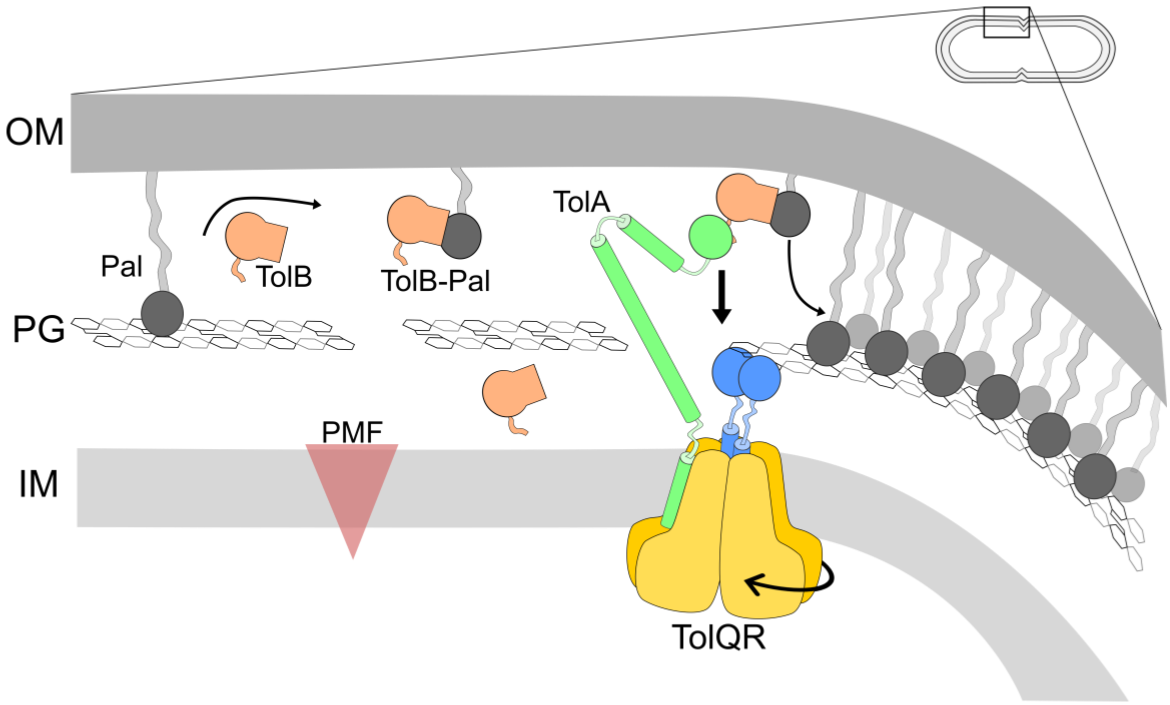

Fig. S1 | The Tol-Pal system uses a mobilisation-and-capture mechanism to accumulate Pal at the division site. Pal is a PG-binding lipoprotein, which tethers the inner leaflet of the OM to the PG layer. The soluble periplasmic protein TolB displaces Pal from the PG, effectively mobilising Pal, which diffuses around the cell. During cell division TolQR and the effector TolA are independently recruited to mid-cell (7, 8), where the TolQRA complex strips TolB from Pal (6, 9). This results in local deposition of Pal, the accumulation of OM-PG tethers and invagination of the OM. Free TolB is recycled, which diffuses through the periplasm to mobilise additional Pal molecules for mid-cell localisation. Figure adapted from Szczepaniak *et al*. and Webby *et al*. (1, 6).

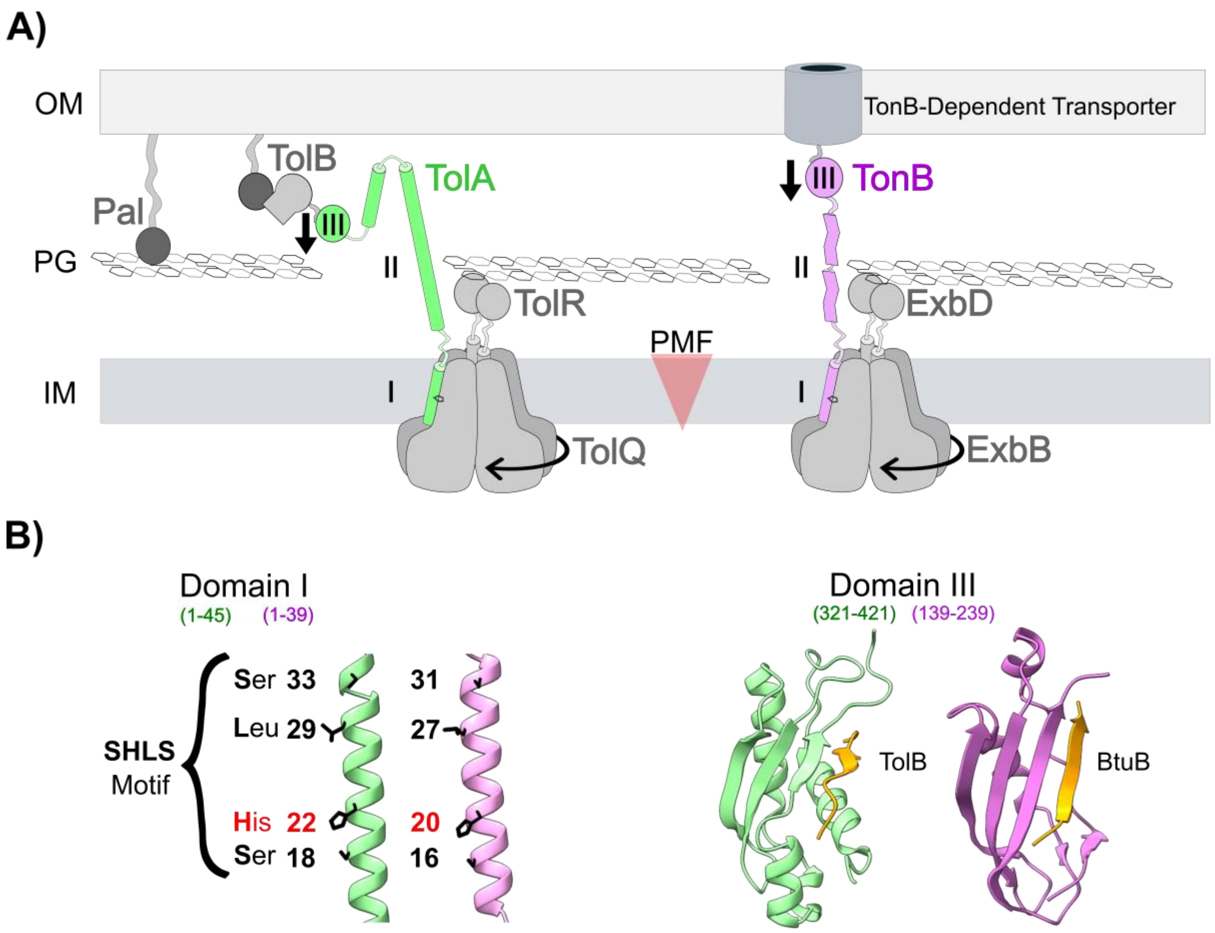

Fig. S2: TolA and TonB have structurally dissimilar central domains that extend through the periplasm. A) Tol and Ton exert tension at the OM by binding OM-targets via β-sheet augmentation. Rotation of the motor complex is postulated to energise TolA/TonB such that rotation (*curved arrow*) is transduced to tension perpendicular to the plane of the membrane (*straight arrow*). The three structured domains of TolA and TonB (I-III) are identified. In TolA, domain II is predicted to form a a coiled-coil while in TonB domain II is composed of two type II polyproline helices (10). B) Structural data and predictions demonstrate that TolA (*green*) and TonB (*pink*) share homologous N- and C- terminal domains. Domain I structural predictions model the SHLS motif in almost identical positions, explaining observations of motor-transducer cross complementarity (10-12). Domain III structures shown are for *Pseudomonas aeruginosa* TolAIII in complex with a peptide derived from the N-terminus of TolB (*orange*), determined by solution NMR (PDB: 6S3W) (6), and *Escherichia coli* TonBIII in complex with the BtuB TonB-box (*orange*) determined by X-ray crystallography (PDB: 2GSK) (13).

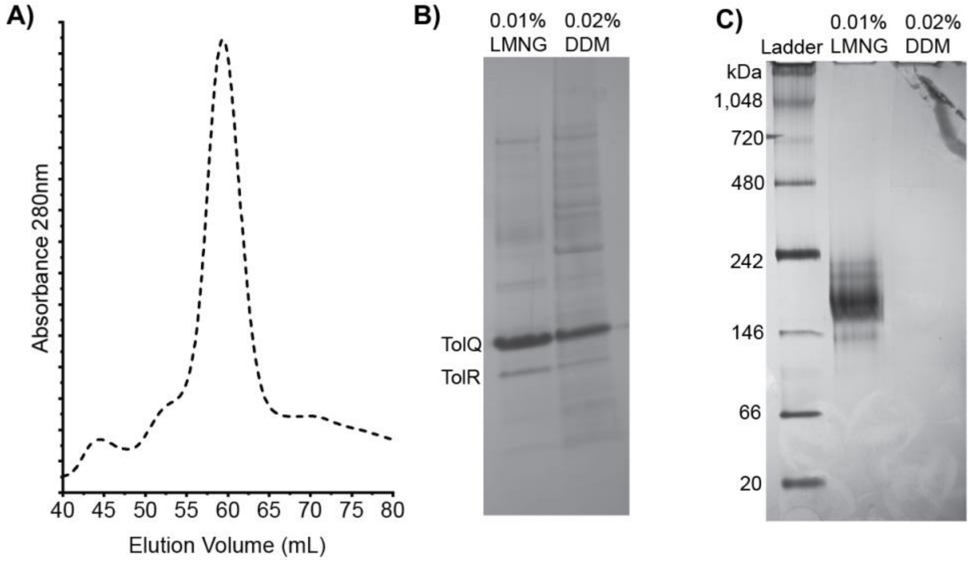

Fig. S3: Isolation and characterisation of the *E. coli* TolQR complex in LMNG. A) Absorbance (280 nm) profile from size-exclusion chromatography (SEC) of TolQR in 0.01% LMNG, pH 7.5, applied to a Superdex200 16/60 column, The majority of protein elutes in the main peak, 56-65 mL. B) SDS-PAGE (10-20% Tris-glycine gel) of TolQR purified through inner membrane extraction, affinity chromatography (His-tag on TolR), and SEC, using detergent based buffers. Bands confirmed to be TolQ and TolR by peptide fingerprinting, were observed in final samples from buffers containing either LMNG of DDM. C) Blue native-PAGE of samples in (B), shows higher order complexes are only detected in TolQR purified in LMNG detergent, despite both LMNG and DDM successfully solubilising each component.

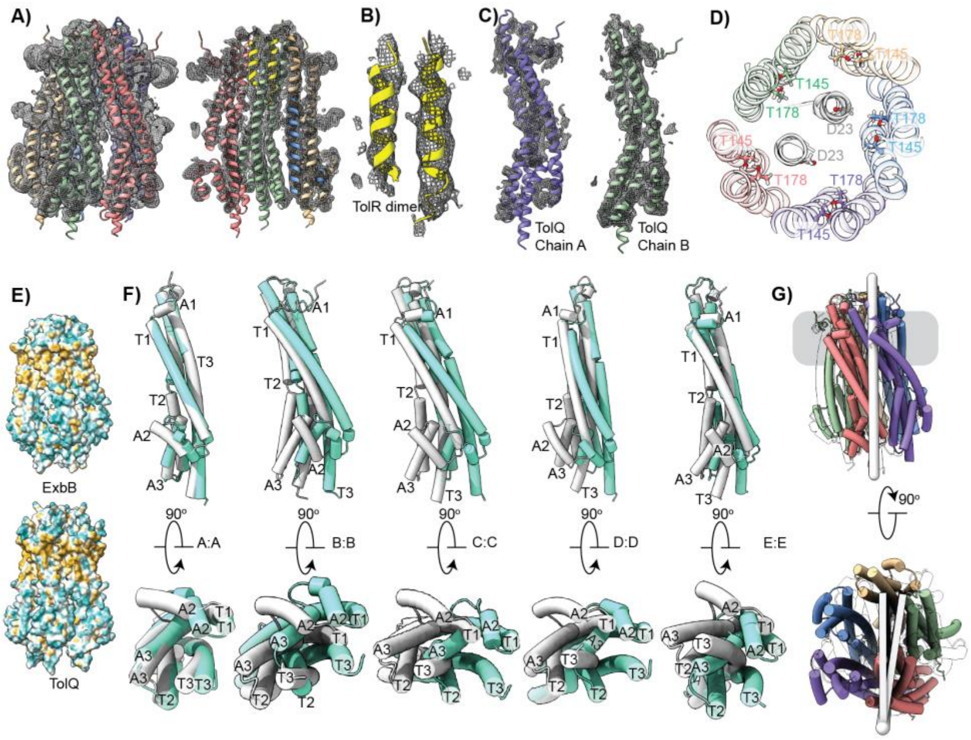

Fig. S4: Comparison of TolQR stator complex with those of ExbBD and MotAB. A) TolQR atomic model (*cartoon*) docked into cryoSPARC refined map (*mesh*), with cut through (*right*) to show the density across the pore. B) TolR dimer (*yellow*) docked into the electron density map generated in cryoSPARC, shows one helix is more resolved than the other. C) Cut-out map to model fits for TolQ chain A (*purple*) and TolQ chain B (*green*). The density for TolQ chain A is poor relative to other chains (B-E), which all resemble that of TolQ chain B. D) Cut through of the top of the TolQR complex with the position of conserved Thr residues (sticks) involved in proton transfer are displayed for each TolQ monomer (*cartoon*). Subunits are represented as independent colours. The position of TolR D23 which also plays a role in proton transduction is shown. E) Surface hydrophobicity profiles calculated in ChimeraX (*yellow*, hydrophobic; *green,* hydrophilic) for ExbB (*left*) and TolQ (*right*) pentameric assemblies. Near-identical hydrophobic belts accommodate the proteins within the inner membrane. A short helix (A1) on the periplasmic side of the protein contributes to the hydrophobic region along with the transmembrane helices (T1-3). F) Alignment of individual monomers of ExbB (*green*) to a TolQ (*grey*). Pentamers were aligned (RMSD of 1.222 Å) on monomer A (*left*) with overlays of every equivalent chain (A-E) shown as they sit when A:A overlay remains fixed. Comparison of monomer A:A overlay shows that the same secondary structures and helical arrangement is adopted by both ExbB and TolQ. Sequential tracking through each monomer set that remain rigidly fixed following alignment of A:A shows that the remainder of the monomers that comprise the pentameric structure of TolQ, are distorted relative to ExbB. The cytoplasm exposed helices particularly T2, T3, A3, and A4 (*SI* movie S1), rotate 0-20° as you track through the discrete subunit pairs (*SI* Table S2). Flipping the side view (*top panel*) by 90° to view from the cytoplasmic side of the structures, further highlights the structural perturbations between monomers that contribute to the respective pentameric complexes of ExbB and TolQ. G) TolQ pentamer coloured by chain with rotation axis (*grey* rod) shown, relative to orientation in the membrane (*grey* rectangle). At 10° rotation (slabs representing start and end point) is required to align chain D (*yellow*) of TolQ model (*coloured by monomer*) to chain D (*white*) of ExbB model (*transparent*). Pentamers were aligned through chain B (*pink*).

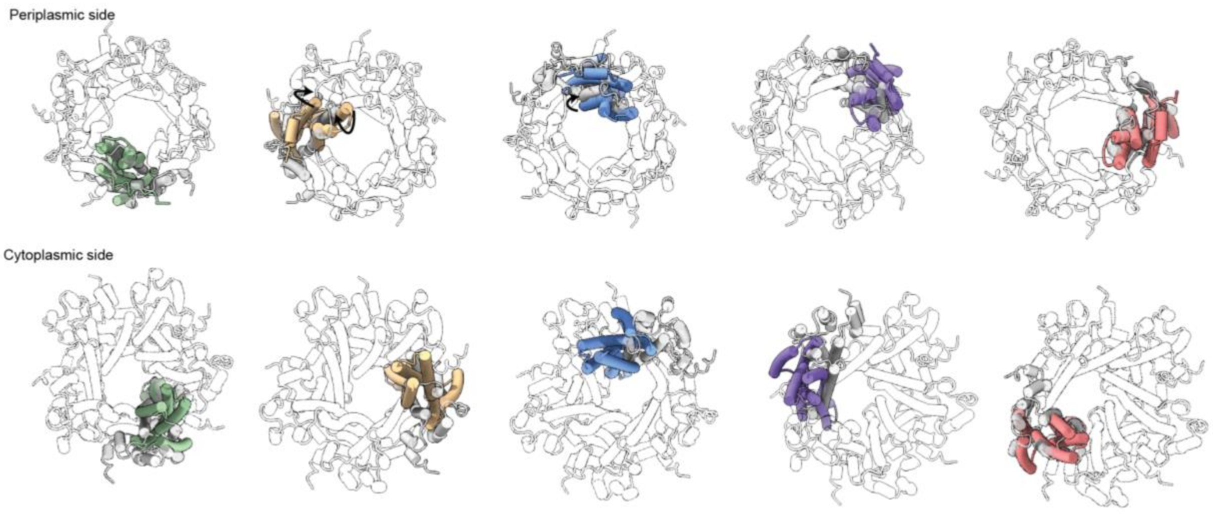

Fig. S5: MotA and TolQ adopt pentamer conformations related by rotation. The MotA (*transparent*) pentamer was aligned with the best fitting chain of TolQ (*green*) in chimeraX. Fit of remaining TolQ monomers (*Coloured*) to MotA (*grey*) are shown sequentially without any further realignment. In all cases the most pronounced distortions between the two proteins are observed in the position of accessory helices on the cytoplasmic side of the pentamer. Only minor perturbations of accessory helices on the periplasmic side and transmembrane helices occur.

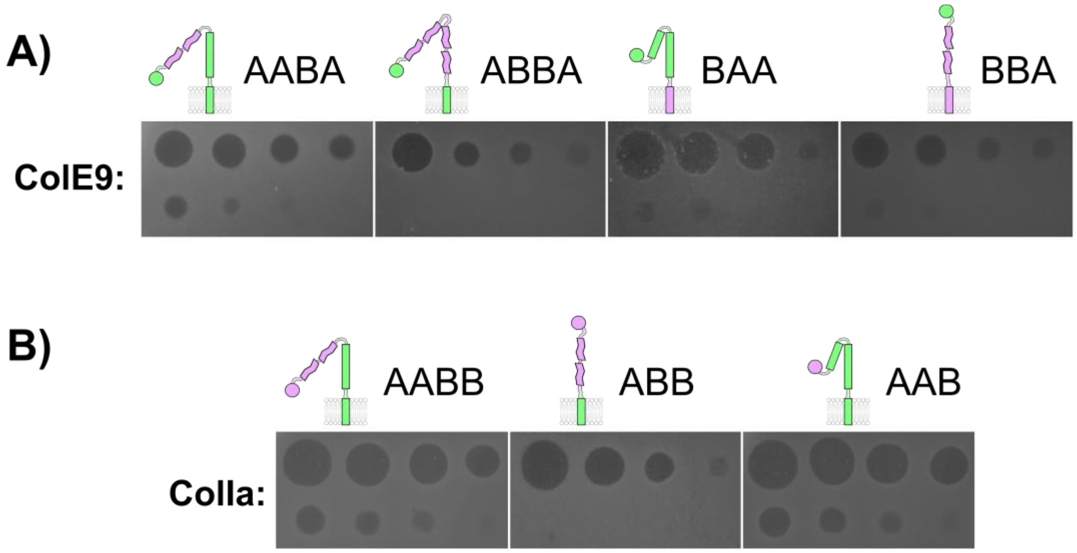

Fig. S6: Cytotoxicity data for additional TolA-TonB chimeric constructs. A) Group A mutants with varying amounts of TonB sequence. Domain swap chimeras of TolA (*green*) and TonB (*pink*) were generated and transformed into *ΔtolA* cells where they restored ColE9 sensitivity. A 4-fold dilution series of purified ColE9 (starting with 8 µM) was applied to a lawn of early-log phase cells. Clearance zones indicate colicin induced killing. BAA was induced with 0.02% arabinose (w/v) due to poor expression (Fig. S9/10). B) Group B mutants with varying quantities of TolA sequence exhibit ColIa sensitivity. Group B constructs were transformed into *ΔtonB* cells, where they restored ColIa sensitivity as indicated by clearance zones. Early log-phase lawns were spotted with a 5-fold series of ColIa (from 10 µM).

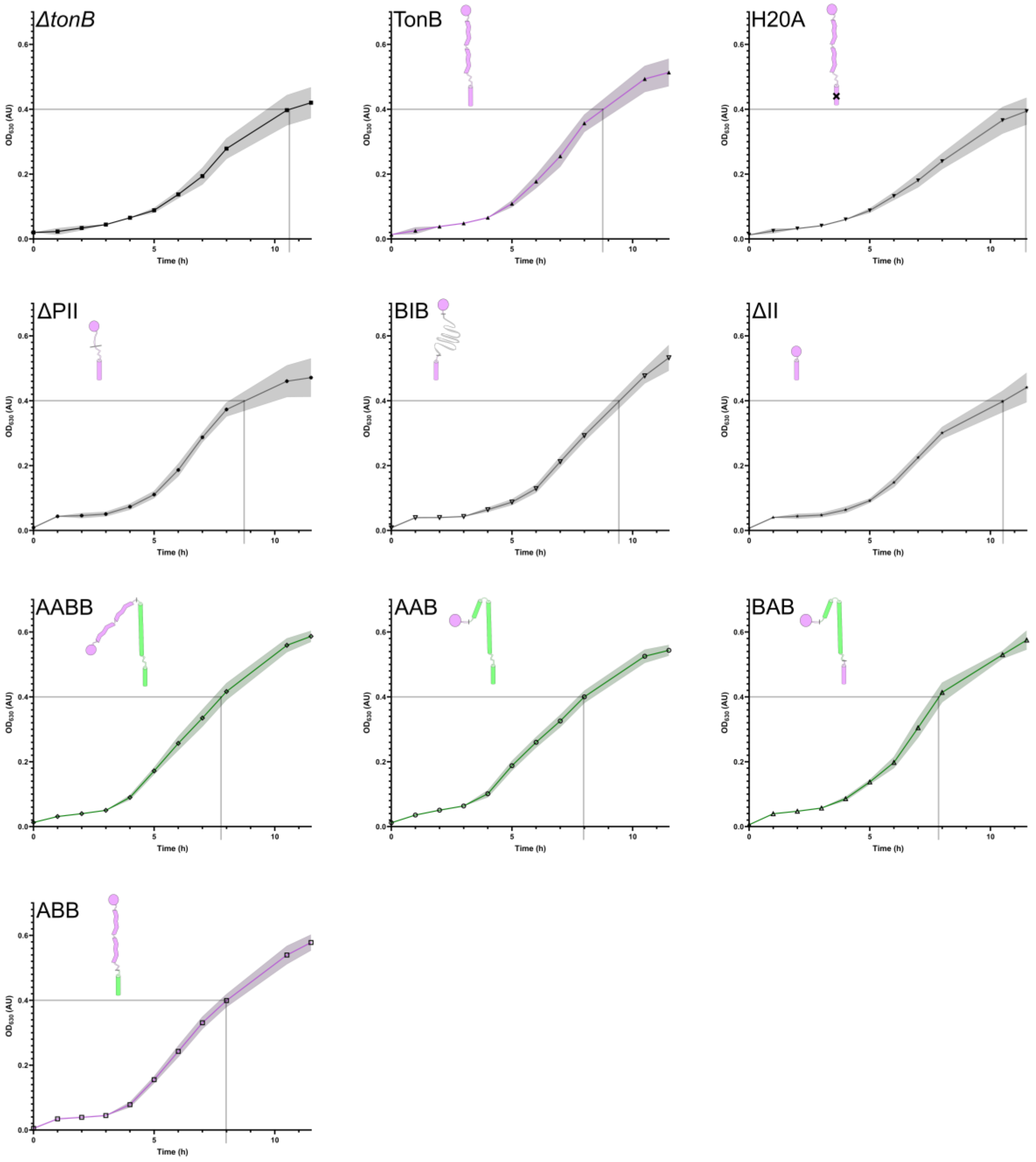

Fig. S7: Group B constructs complement growth in M9 minimal media. Growth curves of Δ*tonB* cells expressing Group B constructs. Mean OD plotted at each time point, with banded regions indicating standard deviation (n=2 biological repeats, comprising 4 technical repeats). Horizontal line at OD = 0.4 accentuates differences in growth rates between the different constructs. ΔPII indicates a construct where the central polyproline region was deleted, while ΔII indicates a construct where the entirety of domain II was replaced with a 7 amino acid residue flexible linker sequence. Green bands indicate a central TolAII, while pink bands indicate central TonBII, and grey bands indicate deletions, IDP replacement and motor-disconnected control.

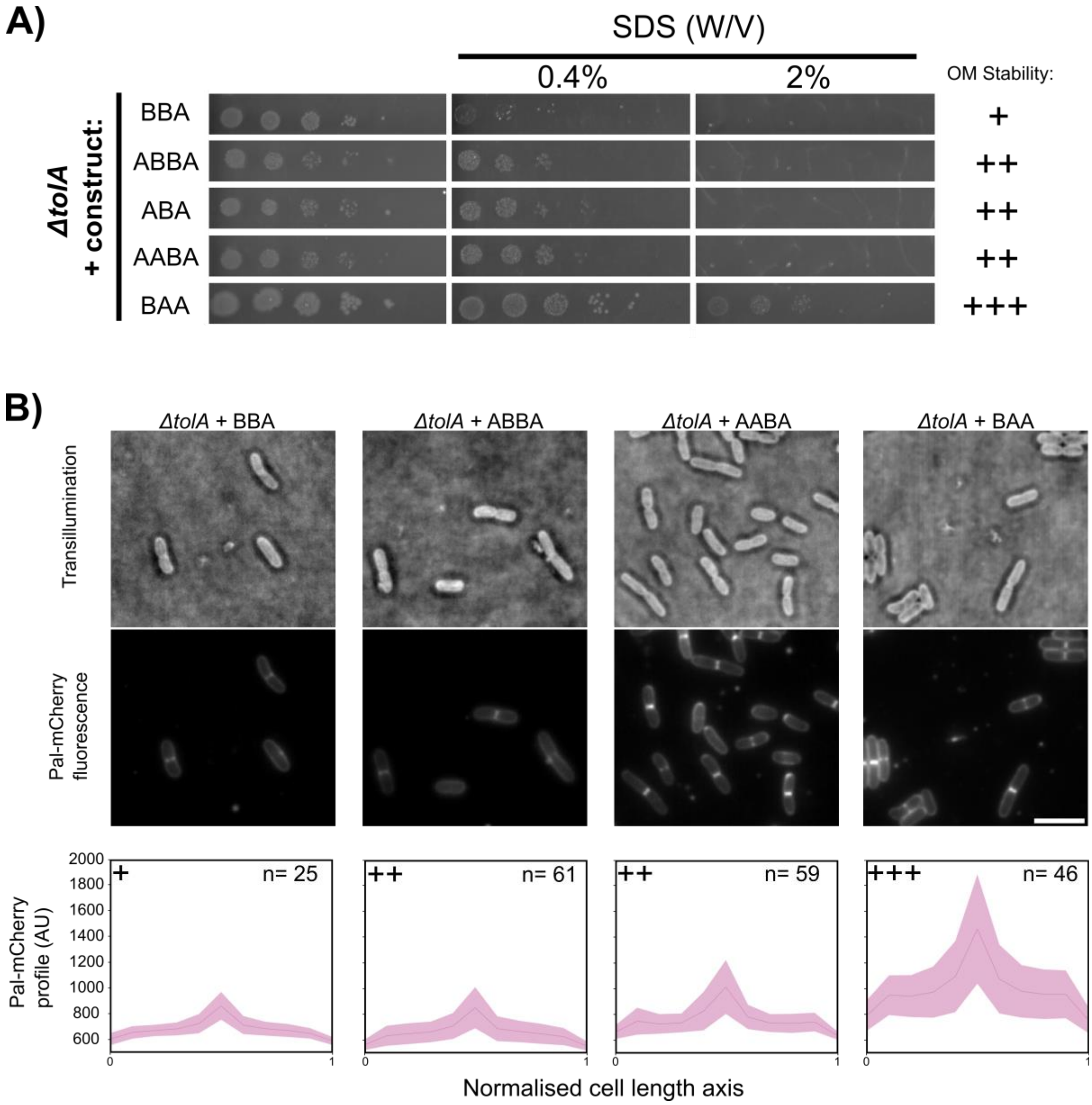

Fig. S8: Additional TolA-like constructs exhibit intermediate OM stabilities, which broadly correlate with the degree of septal Pal localisation. A) Group A constructs exhibit a spectrum of OM stabilities. *ΔtolA* cells were transformed with group A constructs, grown to OD_{600} of 0.5 and plated to assess their capacity for SDS tolerance. Full complementation (wild-type growth), on 2% SDS (w/v) graded "+++", moderate growth on 0.4% SDS (w/v) rated "++", and low growth on 0.4% SDS rated "+". ABA is included, from Fig. 3, for comparison. B) Group A constructs have varied Pal accumulation, which correlates with observed OM stability. *ΔtolA Pal-mCherry* cells were transformed with Group A constructs, grown to mid-log phase, then imaged by transillumination (*top*) and fluorescence microscopy (*centre*). Scale bar, 5 µm. Profiles were obtained as described for Fig. 3B. Central line indicates mean fluorescence intensity along the cell axis as averaged from *n* dividing cells, while banded region indicates a single standard deviation.

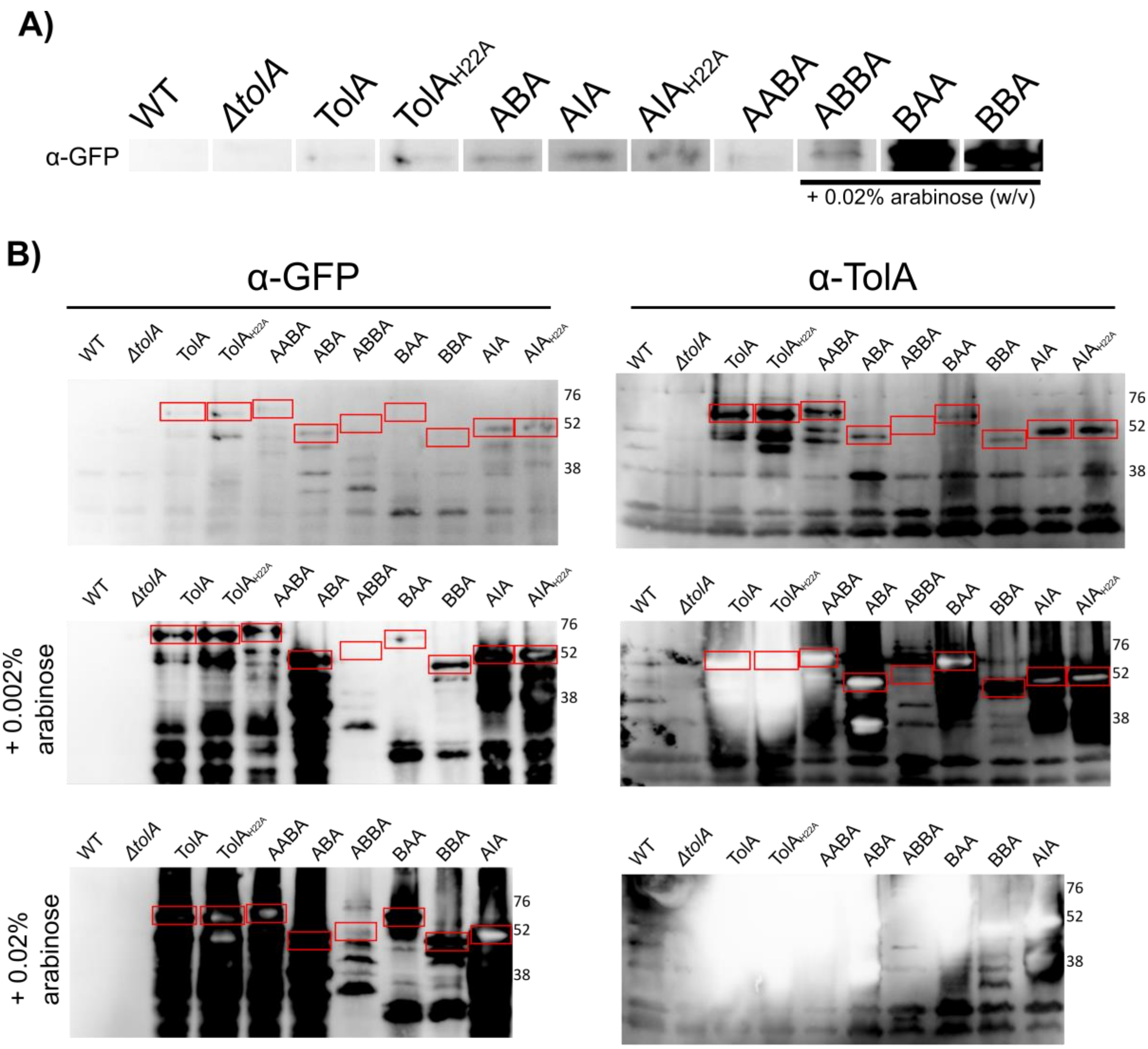

C)

	Name	MW (kDa)
	WT	43.2
	ΔtolA	-
N-terminal GFP fusion	TolA	71.2
	TolA H22A	71.2
	AABA	72.5
	ABA	54.4
	ABBA	64.6
	BAA	74.2
	BBA	51.8
	AIA	53.9
	AIA H22A	53.9

Fig. S9: Western blot of Group A construct expression levels. A) Expression levels of group A constructs from whole cell lysates used in SDS assays (Fig. 3, SI Fig. S8). Equivalent induction levels as shown were used for all microscopy experiments. Polyclonal α-TolA antibodies against TolAII-III were reported previously (14). In case epitopes were not recognised in some TolA-GFP constructs, labelling with α-GFP antibodies were also used. In particular, ABBA was found to express poorly. B) Overview of construct expression at different arabinose concentrations, as labelled by both α-GFP and α-TolA antibodies. Red boxes indicate expected positions of constructs based on mobility. C) Expected molecular weights of each construct.

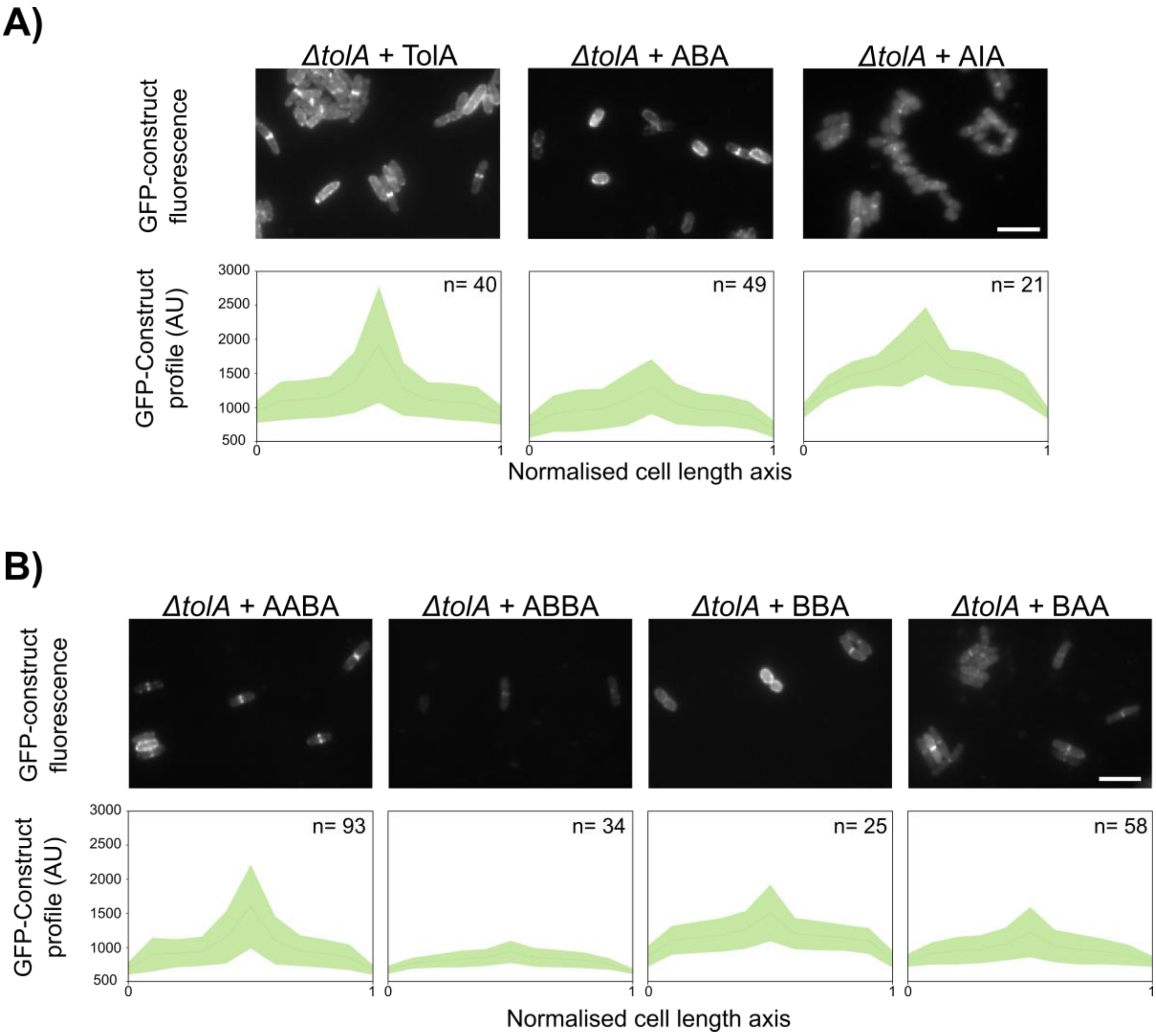

Fig S10: Septal localisation of TolA-GFP constructs. A) Key group A constructs exhibit septal localisation when expressed in *ΔtolA* cells. No arabinose induction was applied to these samples. Fluorescence micrographs were analysed as described for Pal-mCherry profiles previously. Central line indicates mean fluorescence along the cell length axis, and banded regions indicate a single standard deviation for *n* cells. Scale bar indicates 5 µm. B) Additional group A constructs exhibit septal localisation in *ΔtolA* cells. Samples ABBA-GFP, BBA-GFP and BAA-GFP were induced with 0.02% arabinose (w/v). Each image/profile shown is taken from the same cell samples with Pal-mCherry profiles shown previously. Scale bar indicates 5 µm.

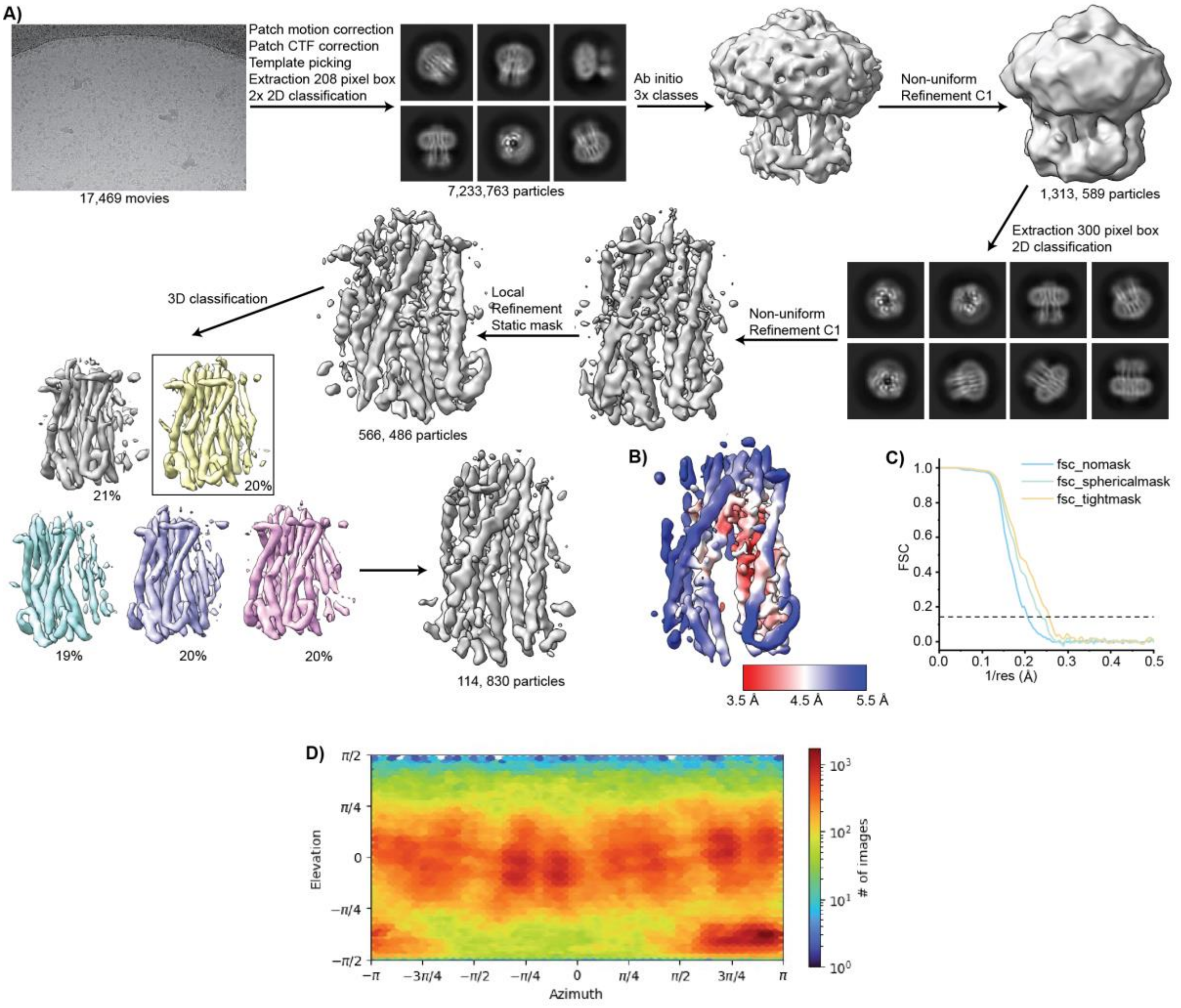

Fig. S11: Cryo-EM workflow. A) Single-particle processing workflow used to generate TolQR structure from micrographs of detergent purified complex. B) Local resolution (calculated in cryoSPARC) mapped on to the final structure. C) Gold-standard FSC curves with 0.143 cut-off for final map generated following local refinement in cryoSPARC. D) Particle distribution profile for final map, generated in cryoSPARC.

Sup movie S1: Structural morph from ExbB pentamer to TolQ pentamer.

ExbBD (6TYI) chain IDs were renamed to match TolQR nomenclature and pentameric structures aligned on chain A within ChimeraX (matchmaker). Structural morph was then generated to track transition required to get from ExbB (start) to TolQ (end).

Sup movie S2: Structural morph from ExbB pentamer to TolQ pentamer to MotA pentamer.

ExbBD (6TYI) and MotAB (6YSL) chain IDs were renamed to match TolQR nomenclature and pentameric structures aligned on chain A within ChimeraX (matchmaker). Structural morph was then generated to track transition required to get from ExbB (start) to TolQ (middle) to MotA (end).

References

1. Webby MN, Williams-Jones DP, Press C, & Kleanthous C (2022) Force-Generation by the Trans-Envelope Tol-Pal System. *Frontiers in microbiology* 13.
2. Meselson M & Yuan R (1968) DNA Restriction Enzyme from E. coli. *Nature* 217(5134):1110-1114.
3. Studier FW & Moffatt BA (1986) Use of bacteriophage T7 RNA polymerase to direct selective high-level expression of cloned genes. *J Mol Biol* 189(1):113-130.
4. Datsenko KA & Wanner BL (2000) One-step inactivation of chromosomal genes in Escherichia coli K-12 using PCR products. *Proc Natl Acad Sci U S A* 97(12):6640-6645.
5. Baba T*, et al.* (2006) Construction of Escherichia coli K-12 in-frame, single-gene knockout mutants: the Keio collection. *Mol Syst Biol* 2:2006 0008.
6. Szczepaniak J*, et al.* (2020) The lipoprotein Pal stabilises the bacterial outer membrane during constriction by a mobilisation-and-capture mechanism. *Nature communications* 11(1):1305.
7. Hale CA, Persons L, & de Boer PAJ (2021) Recruitment of the TolA protein to cell constriction sites in Escherichia coli via three separate mechanisms, and a critical role for FtsWI activity in recruitment of both TolA and TolQ. *Journal of bacteriology*:Jb0046421.
8. Gerding MA, Ogata Y, Pecora ND, Niki H, & de Boer PA (2007) The trans-envelope Tol-Pal complex is part of the cell division machinery and required for proper outer-membrane invagination during cell constriction in E. coli. *Mol Microbiol* 63(4):1008-1025.
9. Petiti M*, et al.* (2019) Tol Energy-Driven Localization of Pal and Anchoring to the Peptidoglycan Promote Outer-Membrane Constriction. *J Mol Biol*.
10. Jumper J*, et al.* (2021) Highly accurate protein structure prediction with AlphaFold. *Nature* 596(7873):583-589.
11. Braun V & Herrmann C (1993) Evolutionary relationship of uptake systems for biopolymers in Escherichia coli: cross-complementation between the TonB-ExbB-ExbD and the TolA-TolQ-TolR proteins. *Mol Microbiol* 8(2):261-268.
12. Koebnik R (1993) The molecular interaction between components of the TonB-ExbBD-dependent and of the TolQRA-dependent bacterial uptake systems. *Mol Microbiol* 9(1):219.
13. Shultis DD, Purdy MD, Banchs CN, & Wiener MC (2006) Outer membrane active transport: structure of the BtuB:TonB complex. *Science* 312(5778):1396-1399.
14. Connolley L, Szczepaniak J, Kleanthous C, & Murray SM (2022) The quantitative basis for the redistribution of immobile bacterial lipoproteins to division septa. *PLOS Computational Biology* 17(12):e1009756.
